# The requirement of p53 for maintaining chromosomal stability during tetraploidization

**DOI:** 10.18632/oncotarget.193

**Published:** 2010-11-24

**Authors:** Chui Chui Ho, Pok Man Hau, Miriam Marxer, Randy Y.C. Poon

**Affiliations:** Division of Life Science, Hong Kong University of Science and Technology, Clear Water Bay, Hong Kong

**Keywords:** cell cycle, genome instability, p53, tetraploidy, transformation

## Abstract

Tetraploidization is believed to promote genome instability and tumorigenesis. Whether tetraploids *per se* are intrinsically unstable and transforming remain incompletely understood. In this report, tetraploidization was induced with cell fusion using mouse fibroblasts. Due to the unequal segregation of chromosomes during multipolar mitosis, the majority of cells were eliminated by p53-dependent mechanisms after tetraploidization. The rare tetraploid fibroblasts that were able to undergo bipolar mitosis remained chromosomally stable and nontransformed over many generations. Suppression of p53 functions during tetraploidization, either by RNA interference or by using p53-deficient mouse fibroblasts, produced cells that were chromosomally unstable. They were fast growing and displayed anchorage-independent growth in soft agar. In contrast, impairment of p53 functions after tetraploids were established was ineffective in triggering chromosomal instability and transformation. Collectively, these results are consistent with a model that during early stages of tetraploidization, the lack of p53 promotes the survival of chromosomally unstable sub-tetraploids, leading to transformation. Once tetraploids are established, however, p53 is not essential for maintaining chromosome stability.

## INTRODUCTION

Limiting genome replication to once per cell cycle is critical for maintaining genome stability. A growing body of evidence indicates that polyploidization can initiate chromosomal instability and transformation [1]. While rereplication is stringently prevented in the normal cell cycle, multiple rounds of genome reduplication, called endoreduplication, occur in cell types such as megakaryocytes, trophoblasts, and plant cells [2]. Polyploidization is also believed to be an essential step during evolution. In his seminal work, Ohno proposed that whole genome duplication provides the primary source of redundant genes for new evolutionary opportunities [3]. Likewise, the multistep progression of cancer – including initiation, progression, heterogeneity, and drug resistance – is a product of evolutionary processes [4]. The extra sets of chromosomes after polyploidization may act as a reservoir of genetic materials to allow clonal evolution of tumor.

The majority of cancer cells are highly aneuploid, displaying dynamic karyotypic changes including gain or loss of whole chromosomes. Tetraploid cells are commonly found in early stages of tumors. Notable examples include Barrett's esophagus [5,6] and cervical carcinoma [7]. Several studies with yeast [8,9] and mammalian cells [10,11] have provided evidence that tetraploidization increases chromosome instability. Moreover, tetraploidy may be an intermediate state in transformation. For example, it was found that during transformation of epithelial cells from mouse salivary glands, tetraploid cells appeared before they undergo a period of chromosome instability and loss [11]. Many viruses can induce tetraploidy via cell fusions. Evidence from *in vitro* and animal models also suggest a link between cell fusion induced by viruses (which then caused tetraploidy) and cancer [12].

A seminal study by Fujiwara *et al.* (2005) indicates that tetraploids can be generated by transiently blocking cytokinesis in p53-null mouse mammary epithelial cells. Importantly, tetraploidization promotes aneuploidy and tumorigenesis [10]. The presence of p53 normally suppresses the generation of tetraploid cells, presumably by activating the intrinsic apoptotic pathway [13]. Several processes that cause tetraploidization, including chromosome nondisjunction (which promotes cleavage furrow regression) [14], persistent telomere damage [15], and virus infection-mediated cell fusion (called heterokaryon) [16] are believed to be important to tumorigenesis. These and other studies provide strong evidence of the importance of tetraploidization as an early step in tumorigenesis.

A p53-dependent “tetraploidy checkpoint” has been proposed to prevent S phase entry in cells that have undergone mitotic slippage or aborted cytokinesis [17]. The checkpoint is believed to sense the increase in chromosome number and halt the cell in a tetraploid G_1_ state. However, the existence of this checkpoint has been disputed [10,18,19]. It is likely that the p53-dependent arrest after tetraploidization is mainly due to DNA damage or centrosomal stress during the aberrant mitosis [2]. Indeed, γ-H2AX can readily be detected in cells undergoing prolonged mitotic arrest [20,21]; even though it is questionable whether the γ-H2AX induced during mitosis is necessary an indicator of DNA damage [22]. Another possibility that has been proposed is that the lack of transcription during mitotic arrest induces stress and triggers the subsequent cell cycle arrest [23].

How tetraploidization promotes chromosome instability remains incompletely understood. At least in yeast, the increase in improper microtubule-kinetochore attachments in tetraploids contributes to chromosome instability [9]. The extra centrosomes after tetraploidization are also critical determinants of chromosome instability [24]. In fact, an increase centrosome number is a common characteristic of several tumors [25]. Because centrosomes are microtubule organization centers, cells with supernumerary centrosomes form multipolar mitotic spindles and display other errors during chromosomal segregation. The uneven segregation of genetic materials into daughter cells results in different fates, including mitotic catastrophe, aneuploidy, and transformation. Nevertheless, multipolar mitosis can be suppressed in the cell either by functional silencing of extra centrosomes or by centrosome clustering [26-28].

Although tetraploidization can promote chromosome instability, there is evidence that suggests tetraploidy is a relatively more stable state than other aneuploidy [2]. Our group also found that cells generated from tetraploidization of Hep3B cells are relatively stable [29]. However, cancer cell lines such as Hep3B do not contain functional p53 and are already aneuploid and transformed before tetraploidization. In this study, we examined whether the tetraploidy state is intrinsically unstable by using untransformed mouse fibroblasts. We found that tetraploid fibroblasts generated by cell fusion are chromosomally stable over many generations, even when p53 is depleted. In contrast, tetraploids induced in the absence of p53 are chromosomally unstable and transformed.

## RESULTS

### Tetraploidization is accompanied with a rapid loss of chromosomal stability

**Figure 1:**
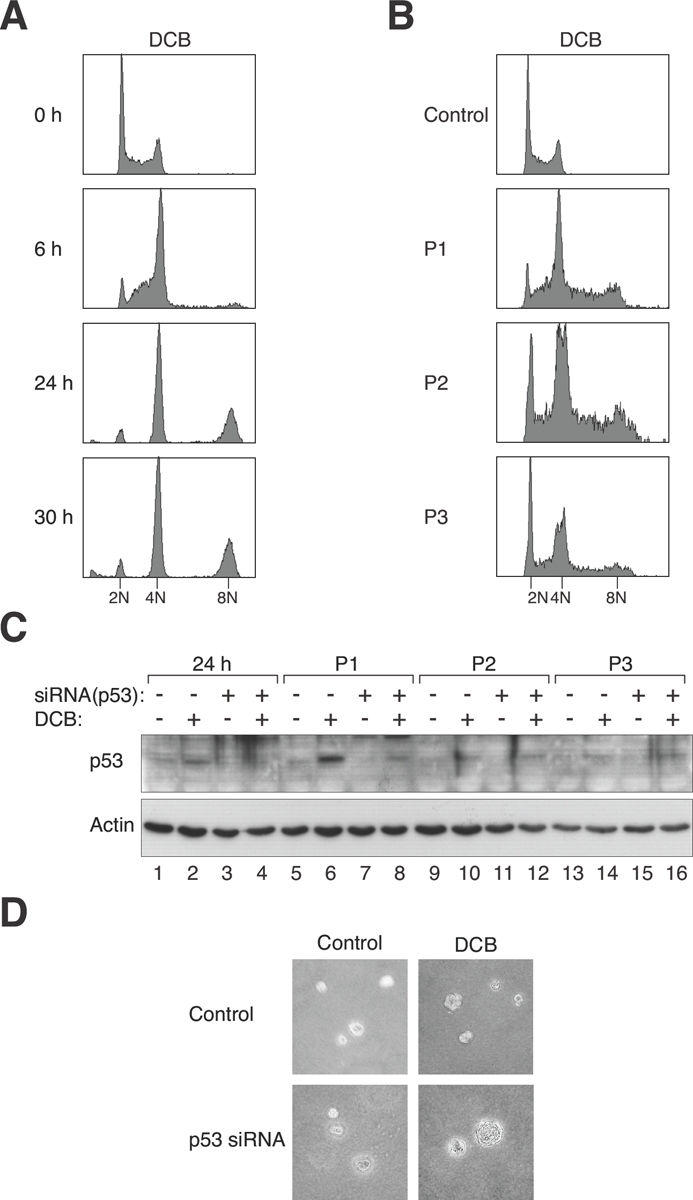
Tetraploidization and p53 depletion induce chromosome instability and transformation **(A) DCB-induced tetraploidization.** Swiss 3T3 fibroblasts were treated with DCB and harvested at the indicated time points for flow cytometry analysis. The positions of 2N, 4N, and 8N DNA contents are indicated. **(B) DCB-induced tetraploids are chromosomally unstable.** Fibroblasts were either mock-treated or incubated with DCB for 24 h. The DCB-treated cells were then harvested at the three subsequent passages (P1, P2, and P3) for flow cytometry analysis. The positions of 2N, 4N, and 8N DNA contents are indicated. **(C) Activation of p53 after DCB-induced tetraploidization.** Fibroblasts were transfected with control or p53 siRNA. The cells were then treated with DCB for 24 h. After washing out the DCB, the cells were propagated in drug-free medium and harvested at three subsequent passages (P1, P2, and P3). Lysates were prepared and analyzed with immunoblotting. **(D) Transformation is induced by DCB in combination with p53 downregulation.** Fibroblasts were transfected with either control or p53 siRNA. At 24 h after transfection, the cells were treated with either buffer or DCB for 24 h. The cells were allowed to recover for three passages and subjected to soft agar transformation assay.

Swiss 3T3 fibroblasts expressing wild type p53 were used to examine genome instability after tetraploidization. Swiss 3T3 were used instead of primary fibroblasts because p53 is frequently mutated when MEFs are immortalized using the 3T3 protocol. To induce tetraploidization, the cells were incubated with dihydrocytochalasin B (DCB), a drug that interferes with actin assembly, to inhibit cytokinesis. Flow cytometry analysis confirmed that DCB-treated cells displayed mainly tetraploid DNA contents relative to untreated cells (Figure 1A). Cells containing up to 8N DNA contents could be detected at 24 h after DCB treatment.

After treatment with DCB for 24 h, the fibroblasts were propagated in drug-free medium. Although tetraploids could be detected at passage 1, they were progressively depleted during subsequent passages (Figure 1B). Immunoblotting of the lysates revealed that p53 was strongly activated after tetraploidization (Figure 1C, lanes 2 and 6). These results suggest that tetraploidization of fibroblasts was associated with an activation of p53 followed by the disappearance of the tetraploids.

To determine if DCB-induced tetraploidization could promote transformation, cells were plated in soft agar to assay for anchorage-independent growth. Figure 1D shows that DCB-treated 3T3 cells were unable to form colonies in soft agar. By contrast, depletion of p53 with three siRNAs (see later for the characterization of the siRNAs) in DCB-treated 3T3 stimulated anchorage-independent growth. As a control, transfection of p53 siRNA in control 3T3 cells did not promote anchorage-independent growth. Immunoblotting verified that the induction of p53 after DCB treatment was suppressed by the siRNAs (Figure 1C). These data are in agreement with the findings of Fujiwara *et al.* (2005) that primary cells can be transformed by a combination of tetraploidization and a loss of p53.

Collectively, these observations suggest that tetraploidy induced in 3T3 fibroblasts by DCB treatment was unstable, and that transformation could be promoted by the downregulation of p53.

### Generation of tetraploid 3T3 fibroblasts by cell fusion

**Figure 2:**
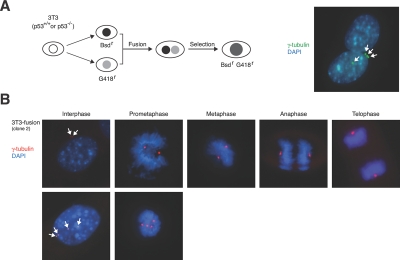
Generation of tetraploids by cell fusion **(A) Fusion of fibroblasts.** Fusion of 3T3 or p53^-/-^ 3T3 fibroblasts was as described in Materials and Methods. Schematic diagram of the cell fusion and selection procedure is shown. At 6 h after fusion, the cells were fixed and centrosomes were visualized with immunostaining for γ-tubulin (arrows). The nuclei were stained with DAPI. **(B) Immunofluorescence microscopy of a representative cell line obtained from fusion of 3T3 cells (clone #2).** Centrosomes were visualized by γ-tubulin staining (arrows). DNA was stained with DAPI. Representative examples of cells from different phases of the cell cycle are shown. While interphase cells containing four centrosomes were frequently observed, prometaphase cells containing multiple centrosomes were observed with low frequency.

To examine the contribution of p53 to genome stability after tetraploidization, p53^-/-^ mouse embryonic fibroblasts were propagated using a 3T3 protocol (called p53^-/-^3T3 herein). Given that most DCB-treated cells loss tetraploidy during passage, more stable tetraploid cells were generated with a strategy involving cell fusion and antibiotic selection (Figure 2A). Plasmids expressing blasticidin- or G418-resistant gene were first transfected individually into 3T3 or p53^-/-^3T3. Stable cell lines resistant to the respective antibiotics were generated after selection. Cell-cell fusion was then performed between serum-starved blasticidin- and G418-resistant cells. Figure 2A verifies that immediately after fusion, some cells contained two nuclei and four centrosomes. The cells were then selected with both blasticidin and G418. More than a dozen of individual colonies from both p53-positive and -negative background were isolated. Data from several representative clones are presented in this paper.

Examination of the cell lines obtained by fusion with indirect fluorescent microscopy revealed that the majority of cells underwent biopolar mitosis (~ 98%). Four centrosomes were frequently observed in interphase cells and also occasionally in prometaphase cells (Figure 2B). However, only rarely were we able to find cells with more than two microtubule organization centers at later stages of mitosis (~ 2% of mitotic cells). These data suggest that despite the presence of multiple centrosomes during interphase, the cells were able to undergo bipolar mitosis, possibly due to mechanisms involving centrosome clustering [25].

**Figure 3:**
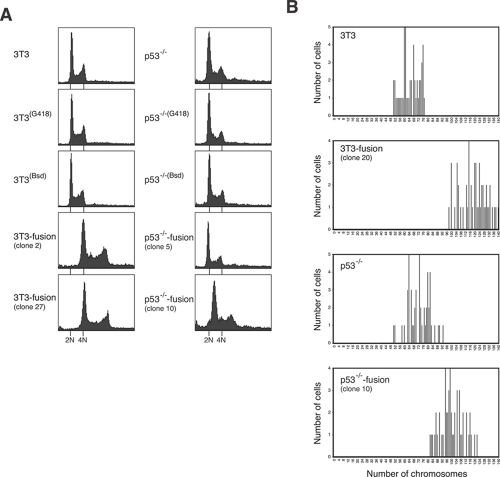
Tetraploidy stability is p53-dependent **(A) Tetraploidy DNA contents can be maintained after cell fusion in p53-containing cells.** Stable cell lines resistant to G418 or blasticidin were generated from 3T3 or p53^-/-^3T3. These cells were fused to generate fusion cell lines. Flow cytometry analysis of the parental cell lines and representative clones of fused cells are shown. The positions of the 2N and 4N DNA contents in relation to the parental cell lines are indicated. **(B) 3T3-fusion cells contain roughly double the chromosome number as the parental 3T3 fibroblasts.** Asynchronously growing 3T3, 3T3-fusion, p53^-/-^3T3 and p53^-/-^3T3-fusion were fixed and processed for chromosome spread. The number of chromosomes was analyzed.

We next examined the DNA contents of the cells obtained by cell fusion. The 3T3-fusion clones contained approximate double the amount of DNA as the parental cells (e.g. clone #2 and #27) (Figure 3A). In marked contrast, most p53^-/-^3T3-fusion clones contained sub-tetraploidy amount of DNA (e.g. clone #5). Some clones did contain more DNA than the parental cells, but they were clearly less than tetraploidy (e.g. clone #10). In agreement with the flow cytometry results, direct counting of chromosomes after mitotic spread indicated that 3T3-fusion cells were roughly tetraploids (Figure 3B).

These results indicate that while fusion of wild type 3T3 fibroblasts generated stable tetraploids, fusion of cells without p53 favored the appearance of sub-tetraploids. These results are consistent with the idea that cells with chromosomal instability following tetraploidization could be eliminated by p53-dependent pathways.

### p53-containing tetraploids are chromosomally stable even after extensive culturing

**Figure 4:**
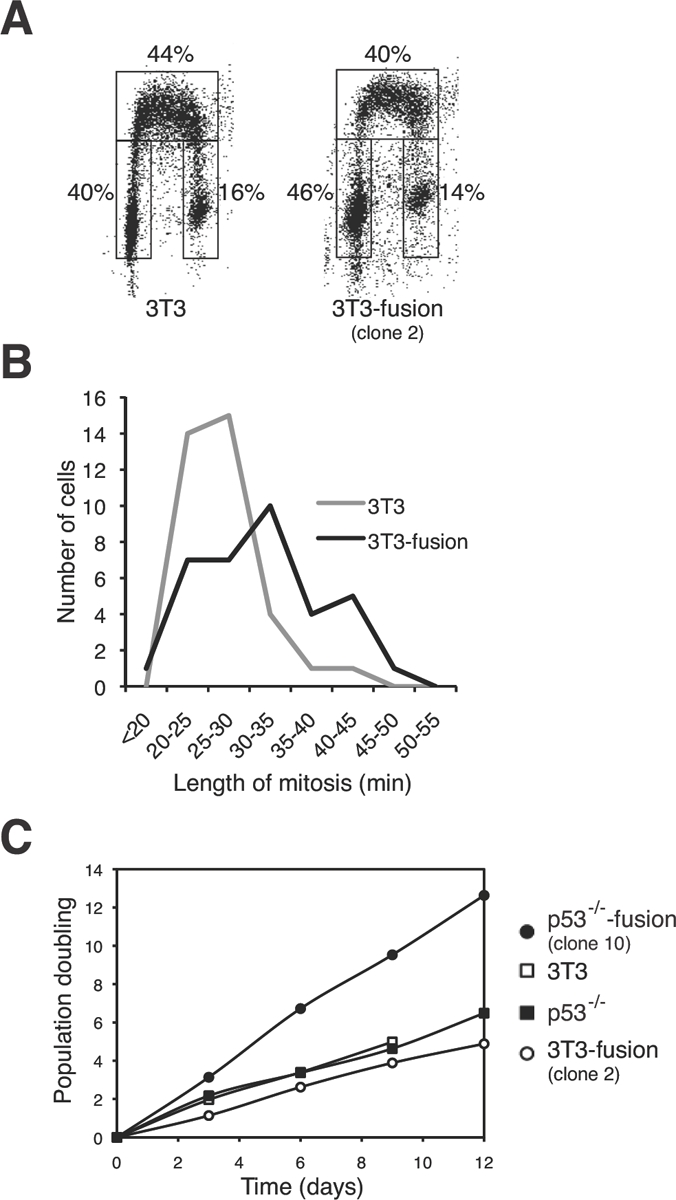
Tetraploidization of p53-containing fibroblasts exerts negligible effects on cell cycle progression **(A) Cell cycle distribution of 3T3 cells is not affected by tetraploidization.** 3T3 or 3T3-fusion cells were pulsed labeled with BrdU. The cells were then processed for BrdU staining and flow cytometry analysis. The percentage of cells in different phases of the cell cycle is displayed. **(B) Tetraploidization increases the mitotic length.** Cells from 3T3-fusion (clone #2) and the parental 3T3 fibroblasts were transfected with a histone H2B-GFP construct. Individual cells were tracked with time-lapse microscopy. The length of mitosis (from DNA condensation to anaphase) of individual cells was measured (n=35). **(C) The growth rate of p53^-/-^3T3 but not 3T3 fibroblasts is significantly enhanced after fusion.** Time-dependent population doubling was measured for the indicated cell lines.

To see if proliferation is affected by tetraploidization, 3T3 and 3T3-fusion were pulse-labeled with BrdU and analyzed by bivariate flow cytometry. Figure 4A shows that the 3T3-fusion cells displayed similar cell cycle distribution as the parental 3T3 cells during the unperturbed cell cycle. To examine mitosis in more detail, cells were transfected with plasmids expressing GFP-tagged histone H2B and tracked with time-lapse microscopy. Figure 4B shows that the duration of mitosis was marginally extended in 3T3-fusion. Finally, the growth rates of 3T3 and 3T3-fusion were also comparable (Figure 4C). In contrast, p53^-/-^3T3-fusion cells proliferated significantly faster than the parental p53^-/-^3T3 (Figure 4C). These results indicate that cells generated by fusion in the absence of p53 displayed proliferation advantage over the diploid counterparts.

**Figure 5:**
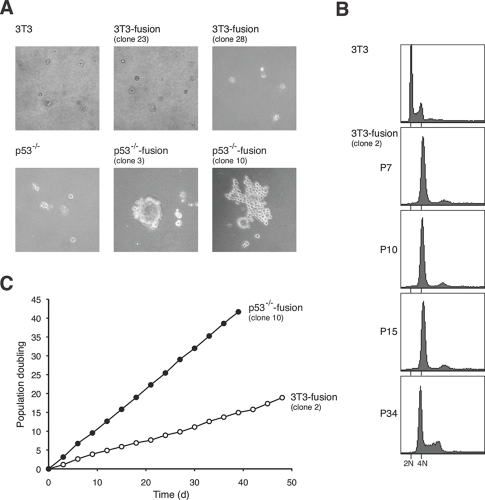
Tetraploids containing p53 are chromosomally stable and not transformed after extensive culturing **(A) Cell fusion induces transformation in cells with p53-negative but not wild type background.** Transformation of the indicated cell lines was assessed by soft agar assays. **(B) Tetraploidy in 3T3 fusion cell lines is maintained after long-term culturing.** The DNA contents of Swiss 3T3 and 3T3 fusion cells collected at different passages were analyzed with flow cytometry. The positions of the 2N and 4N DNA contents in relation to the parental cell lines are indicated. **(C) The growth rate of 3T3 fusion cells does not alter during long-term culturing.** The growth curves of selected clones of p53^-/-^3T3 fusion and 3T3 fusion are shown.

To examine if transformation is promoted by tetraploidization, anchorage-independent growth assays were performed for the various fusion clones. Similar to the parental 3T3 fibroblasts, 3T3-fusion clones were unable to form colonies in soft agar (Figure 5A). In contrast, p53^-/-^3T3-fusion clones could readily proliferate. In agreement with the soft agar assays, cells from p53^-/-^3T3-fusion, but not 3T3-fusion, were able to form tumors after injection into nude mice (data not shown). These data indicate that while cell fusion in the absence of p53 promoted transformation, stable tetraploids generated by fusion of p53-positive fibroblasts remained non-transformed.

Given that 3T3-fusion cells were tetraploids and non-transformed, we next examined whether the tetraploidy state remained stable during long-term culturing. 3T3-fusion cells were carefully cultured with 3T3 protocol for over 100 days (up to passage 34). Figure 5B shows that the tetraploid DNA content was maintained over the time. Similar growth rate was also preserved during the extensive culturing (Figure 5C). Moreover, 3T3-fusion cells from both early and late passages (>30) were unable to grow in soft agar or to induce tumor in nude mice. Collectively, these data indicate that unlike cells from p53^-/-^ background, tetraploids generated from 3T3 were chromosomally stable and remain untransformed over many generations.

### Downregulation of p53 in established tetraploids does not trigger chromosome instability or transformation

**Figure 6:**
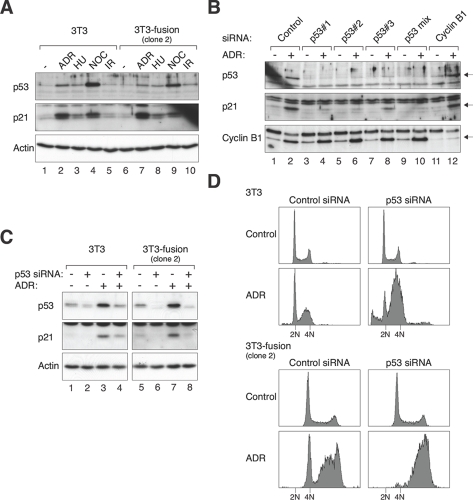
Tetraploid fibroblasts retain functional p53 pathways **(A) The p53 pathway is activated in both 3T3 and tetraploid 3T3 by various stresses.** 3T3 and 3T3-fusion cells were treated with Adriamycin (ADR), hydroxyurea (HU), nocodazole (NOC), or ionizing radiation (IR). After 16 h, the cells were harvested and cell-free extracts were prepared. The expression of p53 and p21^*CIP1/WAF1*^ was detected with immunoblotting. Equal loading of lysates was confirmed with immunoblotting for actin. **(B) Suppression of Adriamycin-induced p53-p21^*CIP1/WAF1*^ responses in 3T3 fibroblasts.** Cells were transfected with control, individual p53 siRNA, a mixture of three p53 siRNAs, or cyclin B1 siRNA. At 24 h after transfection, the cells were either mock-treated or incubated with Adriamycin. After 16 h, the cells were harvested and analyzed with immunoblotting. **(C) Suppression of Adriamycin-induced p53-p21^*CIP1/WAF1*^ responses in 3T3-fusion cells.** 3T3 or 3T3-fusion cells were transfected with either control or p53 siRNAs (mixture). At 24 h after transfection, the cells were either mock-treated or incubated with Adriamycin. After 16 h, the cells were harvested and analyzed with immunoblotting. Actin analysis was included to assess protein loading and transfer. **(D) Depletion of p53 disrupts the G_1_ DNA damage checkpoint in both 3T3 and tetraploid 3T3-fusion.** 3T3 or 3T3-fusion cells were transfected and treated as in panel (C). The cells were then fixed and analyzed with flow cytometry. The positions of the 2N and 4N DNA contents in relation to the 3T3 fibroblasts are indicated.

Given that the absence of p53 during tetraploidization promoted chromosomal instability and transformation, we next examined if downregulation of p53 after tetraploidization could also induce similar effects. To this end, we first validated that both 3T3 and 3T3-fusion indeed contained functional p53. Figure 6A shows that p53 and its downstream target p21^*CIP1/WAF1*^ were induced after treatment with the genotoxic agents Adriamycin and nocodazole. The p53 pathway was also induced to a lesser extent by hydroxyurea and ionizing radiation. These data confirm that 3T3-fusion still retained functional p53.

To downregulate p53, three siRNAs were transfected either individually or in a mixture. Figure 6B shows that the Adriamycin-induced p53 and p21^*CIP1/WAF1*^ in 3T3 fibroblasts could be suppressed by the siRNAs (lanes 4, 6, 8, and 10). As a negative control, depletion of cyclin B1 did not affect the activation of p53-p21^*CIP1/WAF1*^ by Adriamycin (lane 12). Likewise, activation of the p53-p21^*CIP1/WAF1*^ pathway by Adriamycin in 3T3-fusion could be suppressed by the p53 siRNAs (Figure 6C).

To confirm that the DNA damage responses in 3T3 and tetraploid fibroblasts were impaired after p53 depletion, the cells were analyzed with flow cytometry (Figure 6D). While the G1 DNA damage checkpoint is mediated by the p53-p21^*CIP1/WAF1*^ axis, the intra-S and G2 DNA damage checkpoints mainly rely on the ATM/ATR-CHK1/CHK2 axis that is independent on p53 [30]. Flow cytometry analysis revealed the presence of cells in G1, late S phase, and G2 phase following Adriamycin treatment (Figure 6D), indicating the functioning of the checkpoints in both 3T3 and 3T3-fusion fibroblasts. After depletion of p53 with siRNAs, the Adriamycin-mediated G1 delay was abolished in both cell lines. In contrast, the p53-independent intra-S or G2 checkpoints were not affected. Taken together, these data indicate that the genotoxic stress-induced p53 responses are functional in both 3T3 and 3T3-fusion, and that they can be effectively disrupted by siRNAs.

**Figure 7:**
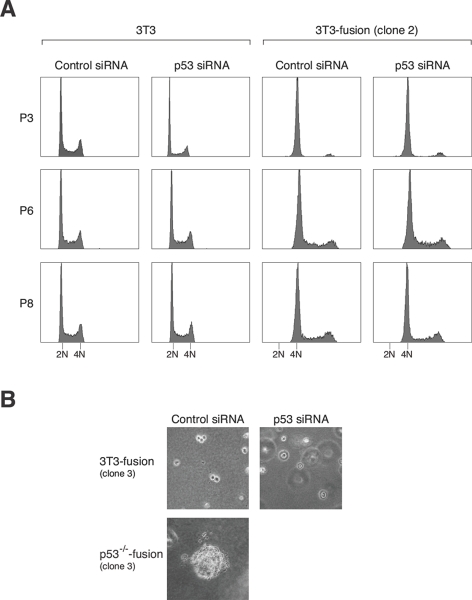
Downregulation of p53 after tetraploidization does not promote transformation **(A) Tetraploidy DNA contents are not affected by p53 depletion.** 3T3 or 3T3-fusion cells were transfected with either control or p53 siRNAs (mixture). The cells were transfected again with the same siRNAs at passages 3 and 6. The DNA contents at the indicated passages were analyzed with flow cytometry. The positions of the 2N and 4N DNA contents in relation to the parental 3T3 fibroblasts are indicated. **(B) Tetraploid 3T3-fusion cells are not transformed by p53 depletion.** Tetraploid 3T3-fusion cells were transfected with either control or p53 siRNAs over the first six passages as in panel (A). After passage 8, transformation was assessed with soft agar assays. Mock-transfected p53^-/-^3T3-fusion cells were used as positive controls.

We then analyzed if downregulation of p53 in 3T3-fusion cells could destabilize the tetraploidy state and lead to transformation. Three rounds of siRNA transfection were performed to ensure that p53 was depleted over several generations. Figure 7A shows that the p53 siRNA treatment did not lead to a collapse of the tetraploidy state. Furthermore, the cells did not acquire transformed phenotype in soft agar (Figure 7B) or nude mice tumorigenicity assays (data not shown). The same results were obtained with cells from early or late passages of two independent 3T3-fusion clones. As a positive control, cells generated from fusion of p53^-/-^3T3 displayed anchorage-independent growth (Figure 7B). Collectively, these results suggest that once tetraploid fibroblasts are stably established, chromosomal stability is no longer sensitive to p53 downregulation.

## DISCUSSION

Unlike organisms such as fish and amphibian, tetraploidization is poorly tolerated in mammals. In fact, only a handful of tetraploid human infants have been documented [31]. On the cellular level, tetraploids are unstable because the extra centrosomes lead to multipolar mitosis, segregating the chromosomes unequally among the daughters. Accordingly, tetraploid fibroblasts generated by transient blocking of cytokinesis with DCB were chromosomally unstable (Figure 1B). In fact, over 80% of mitoses after DCB treatments were multipolar (data not shown). Tetraploids generated by other methods, such as CDK1 inhibitor-promoted mitotic slippage, were similarly unstable (our unpublished data).

Whether tetraploidy *per se* leads to chromosome instability has yet to be established. In this study, we studied tetraploidization by cell fusion of spontaneously immortalized mouse fibroblasts. Although many 3T3 lines are known to contain mutated p53, the Swiss 3T3 cells used here retain functional p53 [32]. This was also confirmed by the activation of both p53 and p21^*CIP1/WAF1*^ after genotoxic stress (Figure 6A).

**Figure 8:**
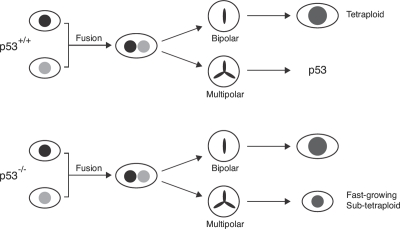
The involvement of p53 in chromosomal instability after tetraploidization Most cells undergo mutipolar mitosis after cell fusion. The unequal segregation of chromosomes leads to the activation of p53-dependent pathway, eliminating the cells by either cell cycle arrest or apoptosis. Cells that are able to undergo bipolar mitosis are chromosomally stable as tetraploids (upper part of the schematic diagram). Subsequent downregulation of p53 in these tetraploids would not affect genome stability. On the other hand, cells that lack p53 during tetraploidization may survive after undergoing multipolar mitosis (lower part of the schematic diagram). Fast growing sub-tetraploids may then be generated.

Based on the results obtained here, we propose a model for p53 in tetraploidization and transformation (Figure 8). Due to the increase in centrosomes after cell fusion, a large population of cells may undergo mutipolar mitosis. The unequal segregation of chromosomes disrupts gene dosage and homeostatsis, possibly leading to the activation of p53-dependent pathways. In agreement with this, p53 was strongly induced during the passages immediately after DCB treatment (Figure 1C). The increase in the duration of mitosis after tetraploidization may also be responsible for inducing p53 [33]. The activation of p53 pathways then eliminated the cells by either cell cycle arrest or apoptosis [13]. Only the rare cells that were able to undergo bipolar mitosis are chromosomally stable as tetraploids and did not activate p53 (Figure 8). Flow cytometry analysis (Figure 3A) and direct chromosome counting (Figure 3B) indicated that the 3T3-fusion cell lines were roughly tetraploidy in relation to the parental 3T3 cells.

How the small number of tetraploid cells could avoid multipolar mitosis is unclear. Many cancer cells also appear to have mechanisms that suppress multipolar mitosis, including functional silencing of extra centrosomes or centrosome clustering [26-28]. In a whole genome-wide RNAi screen with the near-tetraploid *Drosophila* S2 cells, Kwon *et al.* (2008) identified a variety of genes that are required for centrosome clustering, including those function in organizing the spindle microtubules and the spindle-assembly checkpoint [28]. It was also found that the interphase cell shape and adhesion pattern influence the success of the subsequent mitosis in cells with extra centrosomes.

From our model, tetraploids should be chromosomally stable once they are established. In agreement with this, both the chromosome number and the nontransformed phenotype of tetraploid 3T3-fusion were remarkably stable in culture. No discernable change in the ploidy was detected up to passage 34 after clonal isolation (Figure 5B). Moreover, the tetraploid 3T3-fusion cells retained a doubling time similar to the parental cells (Figures 4C and 5C) and were not transformed even after extensive culturing (Figure 5A).

Another characteristic of the model is that the subsequent downregulation of p53 in these tetraploids should not affect genome stability. Accordingly, depletion of p53 after tetraploids had been established did not result in transformation (Figure 7). In contrast, eliminating the p53 responses before tetraploidization by either using siRNAs or a p53^-/-^ background promoted chromosomal instability and transformation.

According to our model (Figure 8), cells that lack p53 during tetraploidization may survive after undergoing multipolar mitosis. Fast growing sub-tetraploids would then be generated and took over the population. Consistent with this idea, transformed p53-/-3T3-fusion fibroblasts exhibited a faster doubling time than the parental cells (Figure 4C). There is already compelling evidence that p53 is involved in guarding against transformation following tetraploidization. Fujiwara *et al.* (2005) indicates that tetraploid p53-null mouse mammary epithelial cells generated by transient blocking of cytokinesis are prone to aneuploidy and tumorigenesis [10]. We also found that induction of tetraploidization of 3T3 fibroblasts with DCB in the presence of p53 siRNA could stimulate transformation (Figure 1D). Likewise, fusion of p53-/-3T3 cells promoted anchorage-independent growth (Figure 5A).

In conclusion, we found that tetraploidy in fibroblasts *per se* is not intrinsically unstable. Cells at early stages of tetraploidization are chromosomally unstable and are probably removed by p53-dependent mechanisms. They are thus prone to transformation in the absence of p53. In contrast, established tetraploids are chromosomally stable and resistant to transformation by depletion of p53.

## METHODS

### Materials

All reagents were obtained from Sigma-Aldrich (St. Louis, MO, USA) unless stated otherwise.

### Cell culture

Swiss 3T3 cells were obtained from the American Type Culture Collection (Manassas, VA, USA). To obtain p53-/- MEFs, pairs of p53+/− mice of C57BL/6 genetic background (JAX Research Systems, Bar Harbor, ME, USA) were mated. Timed pregnant females at 15–16 days of gestation were sacrificed followed by uterine dissection to isolate individual embryos. Embryos were washed thoroughly with PBS, followed by removal of the head and liver. The embryo body was suspended in 2 ml of 0.25% trypsin, and then forced through a 1-cc syringe with an 18-gauge needle. The tissue homogenate was incubated at 37°C for 30 min, triturated by drawing the suspension through a pipette, and then divided into two 100-mm tissue culture dishes. Cells were grown in DMEM medium supplemented with 10% fetal bovine serum (Invitrogen, Carlsbad, CA, USA). A 3T3 subculture schedule was employed by plating 1×106 cells onto each of three 100-mm dishes. Every three days the cells were trypsinized, pooled, and counted to determine the population doublings. The cells were then plated onto three new dishes as before. Unless stated otherwise, cells were treated with the following reagents at the indicated final concentration: Adriamycin (0.2 μg/ml), blasticidin (5 μg/ml), dihydrocytochalasin B (10 μM), G418 (500 μg/ml), hydroxyurea (1.5 mM), nocodazole (0.1 μg/ml). Ionizing radiation was delivered with a caesium137 source from an MDS Nordion Gammacell 1000 Elite Irradiator (Ottawa, Ontario, Canada).

### Cell fusion and stable cell lines

Cells were serum-starved (cultured in DMEM supplemented with 0.1% serum) for 24 h, trypsinized, collected by centrifugation, and resuspended in 5 ml of 50 μM SDS in PBS. After incubation at room temperature for 5 min, the cells were collected by centrifugation and resuspended slowly (over one minute) in 1 ml of polyethylenglycol (PEG) 4000 and incubated for another minute. The cells were then mixed with 9 ml of serum-free medium, collected by centrifugation, washed with serum-free medium, before resuspension in complete medium. Selection medium was added at the second passage after fusion. For G418- and blasticidin-resistant cell lines, cells were transfected with plasmids expressing the respective antibiotic resistant genes. Single colonies were isolated after about two weeks of selection. Antibiotics were not included in the medium once the cell lines were established.

### Transfection and siRNAs

Stealth siRNAs targeting mouse cyclin B1, p53, and control siRNA were obtained from Invitrogen (Carlsbad, CA, USA). Transfection of siRNA and plasmids was carried out using Lipofectamine™ RNAiMAX and Lipofectamine™ 2000 (Invitrogen), respectively.

### Anchorage-independent growth and tumorigenicity assays

Approximately 10^5^ cells were seeded in 3 ml of 0.35% low melting point agarose in growth media. The cell suspension was casted onto 60-mm plates with 3 ml of 0.5% agarose in growth media as an underlay. Photographs were taken 14 days after plating. The experimental protocol for tumorigenicity was evaluated and approved by the Animal Care Committee at HKUST. Six to eight week old nude mice (Balb/cnu) were maintained in pathogen-free conditions. Cells (2×10^6^) in 100 μl of PBS were injected subcutaneously into an area overlying the hind flank. Mice were regularly checked for tumor formation and sacrificed when tumors reached 10–12 mm in diameter, or after 16 weeks of monitoring.

### Mitotic spread

Cells were treated with 0.1 μg/ml of nocodazole for 2 h. Following trypsinization, the cells were resuspended in 3 ml of 75 mM KCl at 37°C for 20 min, then fixed with 3 ml of Carnoy's fixative (3:1 methanol:glacial acetic acid). After incubation at room temperature for 30 min, the cells were collected by centrifugation and resuspended in fixative. The cell suspension was dropped onto glass slides, air dried, and stained with Giemsa stain for 15 min before imaging.

### Cell imaging

Live cells imaging was performed using a TE2000E-PFS inverted fluorescent microscope (Nikon, Tokyo, Japan) equipped with a SPOT BOOST™ EMCCD camera (Diagnostic Instrument, Sterling Heights, MI, USA) and a INU-NI-F1 temperature, humidity and CO_2_ control system (Tokai Hit, Shizuoka, Japan). Data acquisition and analysis were carried out using the Metamorph 7.5 software (Molecular Devices, Downingtown, PA, USA). Indirect immuno-fluorescent microscopy was performed as described previously [34]. FITC- and TRITC-conjugated secondary antibodies were obtained from DAKO (Glostrup, Denmark).

### BrdU labeling and flow cytometry

BrdU incorporation, propidium iodide staining, and flow cytometry analysis were performed as described previously [35].

### Antibodies and immunological methods

Immunoblotting was performed as previously described [36]. Monoclonal antibody against β-actin was obtained from sources as described previously [37]. Monoclonal antibodies against cyclin B1 (sc-245), γ-tubulin (sc-17787), polyclonal antibodies against p21CIP1/WAF1 (sc-397) and p53 (sc-6243) were obtained from Santa Cruz Biotechnology (Santa Cruz, CA, USA).
